# Drug-related side effects and adverse reactions in the treatment of migraine: a bibliometric and visual analysis

**DOI:** 10.3389/fneur.2024.1342111

**Published:** 2024-02-06

**Authors:** Shijie Wei, Hao Lv, Dianhui Yang, Lili Zhang, Xuhao Li, Yike Ning, Yu Tang, Xinyu Wu, Jing Han

**Affiliations:** ^1^School of Acupuncture and Tuina, Shandong University of Traditional Chinese Medicine, Jinan, China; ^2^Affiliated Hospital of Shandong University of Traditional Chinese Medicine, Jinan, China; ^3^Institute of Acupuncture and Moxibustion, Shandong University of Traditional Chinese Medicine, Jinan, China

**Keywords:** bibliometric analysis, visualization, migraine, drug-related side effect, adverse

## Abstract

**Background:**

Migraine imposes a substantial global burden, impacting patients and society. Pharmacotherapy, as a primary treatment, entails specific adverse reactions. Emphasizing these reactions is pivotal for improving treatment strategies and enhancing patients’ well-being. Thus, we conducted a comprehensive bibliometric and visual analysis of relevant literature.

**Methodology:**

We conducted a comprehensive search on the Science Citation Index Expanded within the Web of Science, restricting the literature for analysis based on criteria such as document type, publication date, and language. Subsequently, we utilized various analytical tools, including VOSviewer, Scimago Graphica, the R package ‘bibliometrix’, CiteSpace, and Excel programs, for a meticulous examination and systematic organization of data concerning journals, authors, countries/regions, institutions, keywords, and references.

**Results:**

By August 31, 2023, the literature was distributed across 379 journals worldwide, authored by 4,235 individuals from 1726 institutions. It featured 2,363 keywords and 38,412 references. ‘HEADACHE’ led in publication count, with ‘SILBERSTEIN S’ as the most prolific author. The United States ranked highest in publication volume, with ‘UNIV COPENHAGEN’ leading among institutions.

**Conclusion:**

Our research findings indicate that researchers in the field continue to maintain a focus on the calcitonin gene-related peptide (CGRP) system and explore diverse mechanisms for drug development through the application of novel biotechnological approaches. Furthermore, it is imperative to enhance the assessment of clinical trial outcomes, consistently monitor the efficacy and safety of prominent drugs such as Erenumab and Fremanezumab. There is a need for further evaluation of acute and preventive treatments tailored to different populations and varying types of migraine.

## 1 Introduction

Migraine, a highly prevalent neurological disorder, affects over 15% of the global population and stands as the second leading cause of disability (1). Migraine imposes a significant burden, with estimated annual treatment costs in Europe reaching approximately €111 billion. The *per capita* cost is approximately €1,222 per year, with 92% attributed to indirect costs stemming from absenteeism and reduced productivity (2). Clinically, it is characterized by recurrent, pulsating, unilateral, moderate-to-severe headaches lasting 4 to 72 h, often accompanied by symptoms such as nausea, vomiting, photophobia, and phonophobia (3). The intricate pathophysiological mechanisms of migraine involve the significant involvement of the trigeminovascular system, gaining widespread attention (4). Additionally, various factors, including hormonal influences (5) and genetic predispositions (6), are believed to play a crucial role in the progression of migraine. Presently, drug therapy remains the cornerstone of migraine treatment, encompassing opioids, triptans, CGRP receptor antagonists, antidepressants, antiepileptic drugs, nonsteroidal anti-inflammatory drugs, among others (7). Among these, preventive medications encompass tricyclic antidepressants, antiepileptics, beta-blockers, and others. In the category of acute (abortive) drugs, triptans and nonsteroidal anti-inflammatory drugs are typically favored as first-line options, while opioids are generally considered a last resort (8). However, the use of these medications may also result in adverse effects, and certain side effects may occasionally persist even after discontinuation of preventive therapy (9). While some side effects may manifest as mild drowsiness or nausea, others can lead to severe complications compromising patient well-being. Therefore, conducting clinical research on drug-related adverse reactions, refining adverse reaction reporting standards in clinical treatment and research, and gaining a comprehensive understanding of the severity and nature of adverse effects hold significant clinical importance.

Over the past few decades, research pertaining to Drug-Related Side Effects and Adverse Reactions in the treatment of migraine has witnessed continual development. A comprehensive understanding of the overall landscape and research trends in this field is of paramount significance. Bibliometrics, as a method for the holistic analysis of publications within a specific domain, plays a pivotal role in enabling researchers to grasp an overview of research within the field (10–12). However, we have not identified any bibliometric studies specifically focusing on Drug-Related Side Effects and Adverse Reactions in migraine treatment. Consequently, these medication-related adverse effects merit our earnest attention.

Therefore, we conducted a thorough assessment of relevant literature within the field through bibliometric analysis and visualization. This involved synthesizing information related to publications, journals, authors, countries, institutions, keywords, and cited references. In doing so, we have delineated the developmental trajectory of pertinent research and speculated on future research directions. We firmly believe that this study will facilitate researchers in comprehending the evolution of the research domain, and aid scholars in embarking on new investigations to make novel discoveries.

## 2 Methods

### 2.1 Data collection

On September 20, 2023, we conducted a literature search in The Web of Science (WOS) Science Citation Index Expanded (SCI-Expanded) database.^1^ The search formula used was as follows: (TS = (Drug-Related Side Effects and Adverse Reactions) OR TS = (Drug-Related Side Effects and Adverse Reactions) OR TS = (Drug Related Side Effects and Adverse Reactions) OR TS = (Drug-Related Side Effects and Adverse Reaction) OR TS = (Drug Related Side Effects and Adverse Reaction) OR TS = (Drug Side Effects) OR TS = (Drug Side Effect) OR TS = (Effects, Drug Side) OR TS = (Side Effect, Drug) OR TS = (Side Effects, Drug) OR TS = (Adverse Drug Reaction) OR TS = (Adverse Drug Reactions) OR TS = (Drug Reaction, Adverse) OR TS = (Drug Reactions, Adverse) OR TS = (Reactions, Adverse Drug) OR TS = (Adverse Drug Event) OR TS = (Adverse Drug Events) OR TS = (Drug Event, Adverse) OR TS = (Drug Events, Adverse) OR TS = (Side Effects of Drugs) OR TS = (Drug Toxicity) OR TS = (Toxicity, Drug) OR TS = (Drug Toxicities) OR TS = (Toxicities, Drug) AND (TS = (Migraine Disorders) OR TS = (Disorder, Migraine) OR TS = (Disorders, Migraine) OR TS = (Migraine Disorder) OR TS = (Migraine) OR TS = (Migraines) OR TS = (Migraine Headache) OR TS = (Headache, Migraine) OR TS = (Headaches, Migraine) OR TS = (Migraine Headaches) OR TS = (Acute Confusional Migraine) OR TS = (Acute Confusional Migraines) OR TS = (Migraine, Acute Confusional) OR TS = (Migraines, Acute Confusional) OR TS = (Status Migrainosus) OR TS = (Hemicrania Migraine) OR TS = (Hemicrania Migraines) OR TS = (Migraine, Hemicrania) OR TS = (Migraines, Hemicrania) OR TS = (Migraine Variant) OR TS = (Migraine Variants) OR TS = (Variant, Migraine) OR TS = (Variants, Migraine) OR TS = (Sick Headache) OR TS = (Headache, Sick) OR TS = (Headaches, Sick) OR TS = (Sick Headaches) OR TS = (Abdominal Migraine) OR TS = (Abdominal Migraines) OR TS = (Migraine, Abdominal) OR TS = (Migraines, Abdominal) OR TS = (Cervical Migraine Syndrome) OR TS = (Cervical Migraine Syndromes) OR TS = (Migraine Syndrome, Cervical) OR TS = (Migraine Syndromes, Cervical) OR TS = (Migraine Disorders). We delimited the time frame up to August 31, 2023, restricting publication types solely to English articles and reviews.

### 2.2 Data analysis and visualization

Two researchers independently reviewed the included literature, manually correcting spelling errors and consolidating overlapping items into singular elements. In cases of disagreement, a third researcher was consulted to reach a consensus.

This study utilized VOSviewer (version 1.6.19) (13), Scimago Graphica (version 1.0.35) (14), the R package “bibliometrix” (version 4.3.1)^2^ (15), CiteSpace (version 6.2.R4) (16), and Microsoft Office Excel 2021 for bibliometric analysis and visualization. VOSviewer and Scimago Graphica were employed for co-authorship and co-occurrence analysis. CiteSpace was utilized for reference and keyword analysis. The R package “bibliometrix” was used for calculating relevant bibliometric indicators such as the number of publications (NP), the number of citations (NC), h-index, g-index, and for visualizing the yearly publication output of journals/authors, along with historiographic analysis. Microsoft Office Excel 2021 was utilized for the comprehensive compilation of the relevant data.

## 3 Results

### 3.1 General analysis of publication status

**Figure 1:**
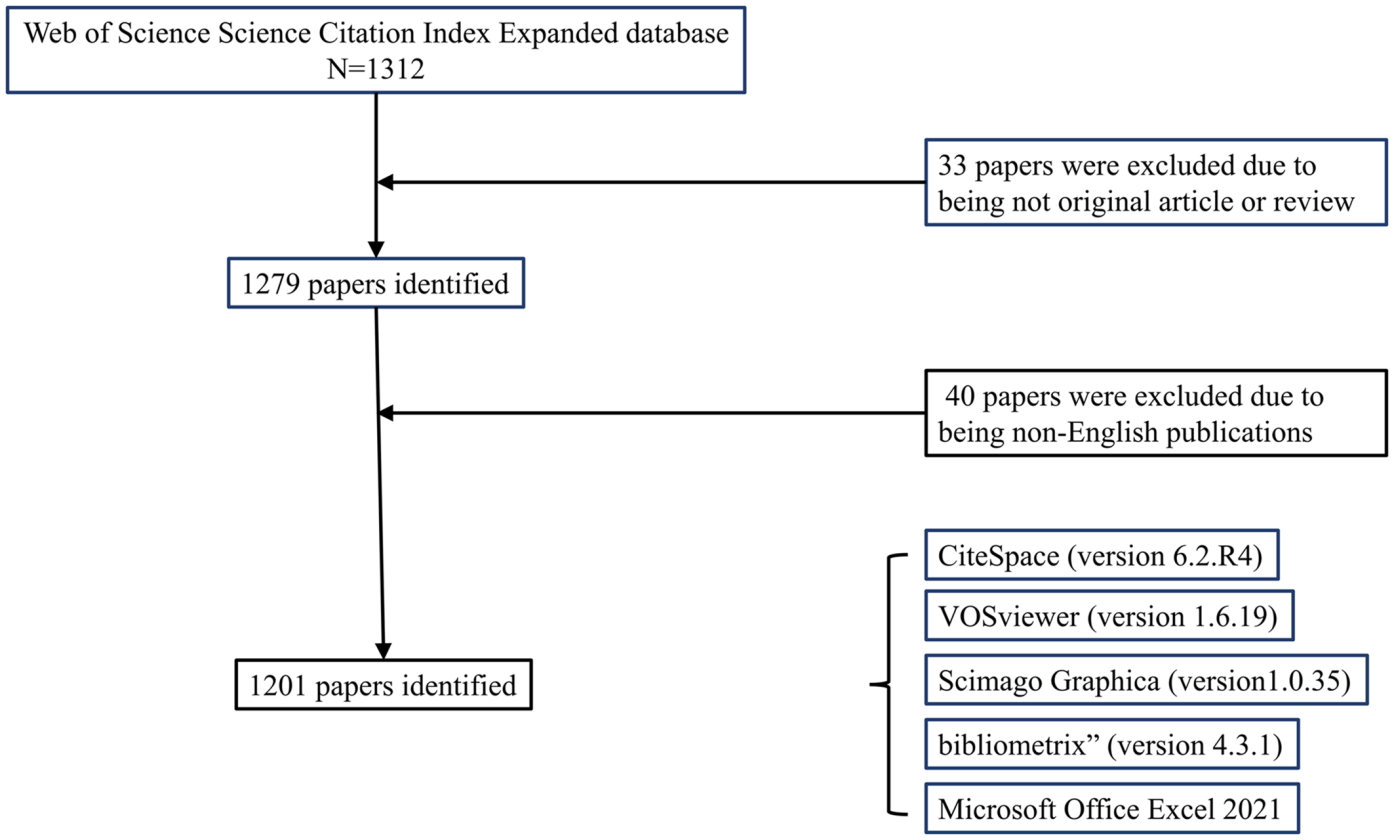
Flowchart of literature screening.

According to the retrieval strategy (Figure 1), a total of 1,312 publications were identified as of August 31, 2023. After excluding other types of articles, 1,279 articles and reviews remained. Following the exclusion of non-English publications, 1,201 items were included in the study. These publications span 60 countries/regions, 379 journals, involve 4,235 authors, and are affiliated with 1726 institutions.

### 3.2 Times cited and publications over time

**Figure 2:**
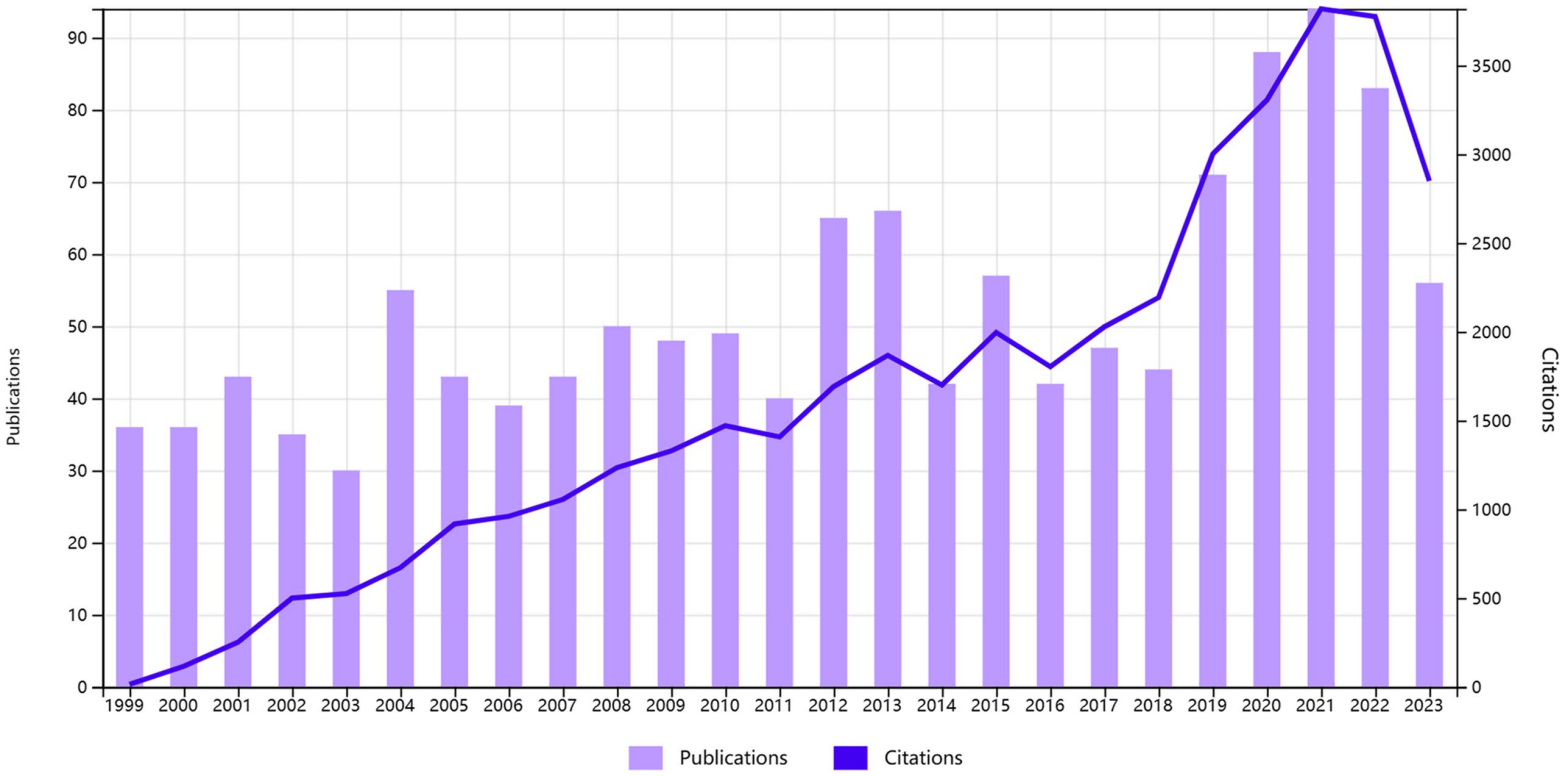
Year of publication and citation.

We depict the distribution of Times Cited and Publications in Figure 2. The Times Cited exhibits a discernible growth trend, reaching its peak at 3841 citations in 2021. The number of publications per year demonstrates that, from 1999 to 2018, the majority of the years maintained a publication count ranging between 30 and 40, with 2012, 2013, and 2015 exceeding 50 publications. The years 2019 to 2021 show a marked upward trajectory, with the number of publications reaching its maximum at 96 in 2021. Subsequently, both Times Cited and Publications have been sustained at a significantly elevated level.

### 3.3 Article analysis

Collectively, these publications have garnered 40,165 citations, with an average of 33.44 citations per article and an h-index of 86. Notably, eight publications have received over 400 citations each. Topping the list, “Calcitonin gene-related peptide receptor antagonist BIBN 4096 BS for the acute treatment of migraine” has been cited the most, accumulating a total of 989 citations. This article substantiates the significant therapeutic efficacy and safety of the CGRP Receptor Antagonist BIBN 4096 BS in the acute treatment of migraines through an international, multicenter, double-blind randomized clinical trial (17). Following closely is the publication titled “Oral triptans (serotonin 5-HT(1B/1D) agonists) in acute migraine treatment: a meta-analysis of 53 trials,” with a cumulative citation count of 728. The authors analyzed 53 clinical trials involving 24,089 patients to observe the efficacy and good tolerability of oral triptans as selective serotonin 5-HT(1B/1D) agonists in the treatment of acute migraines (18). Furthermore, the publication titled “Topiramate for migraine prevention: a randomized controlled trial” has garnered a total of 533 citations. Through a 26-week, randomized, double-blind, placebo-controlled study across 52 North American clinical centers, the authors concluded that topiramate demonstrates significant preventive effects against migraines but may result in side effects such as paresthesia, fatigue, and nausea (19). Collectively, these highly cited publications have accrued over 2,100 citations, each exceeding 500 citations.

#### 3.3.1 Journal analysis

**Figure 3:**
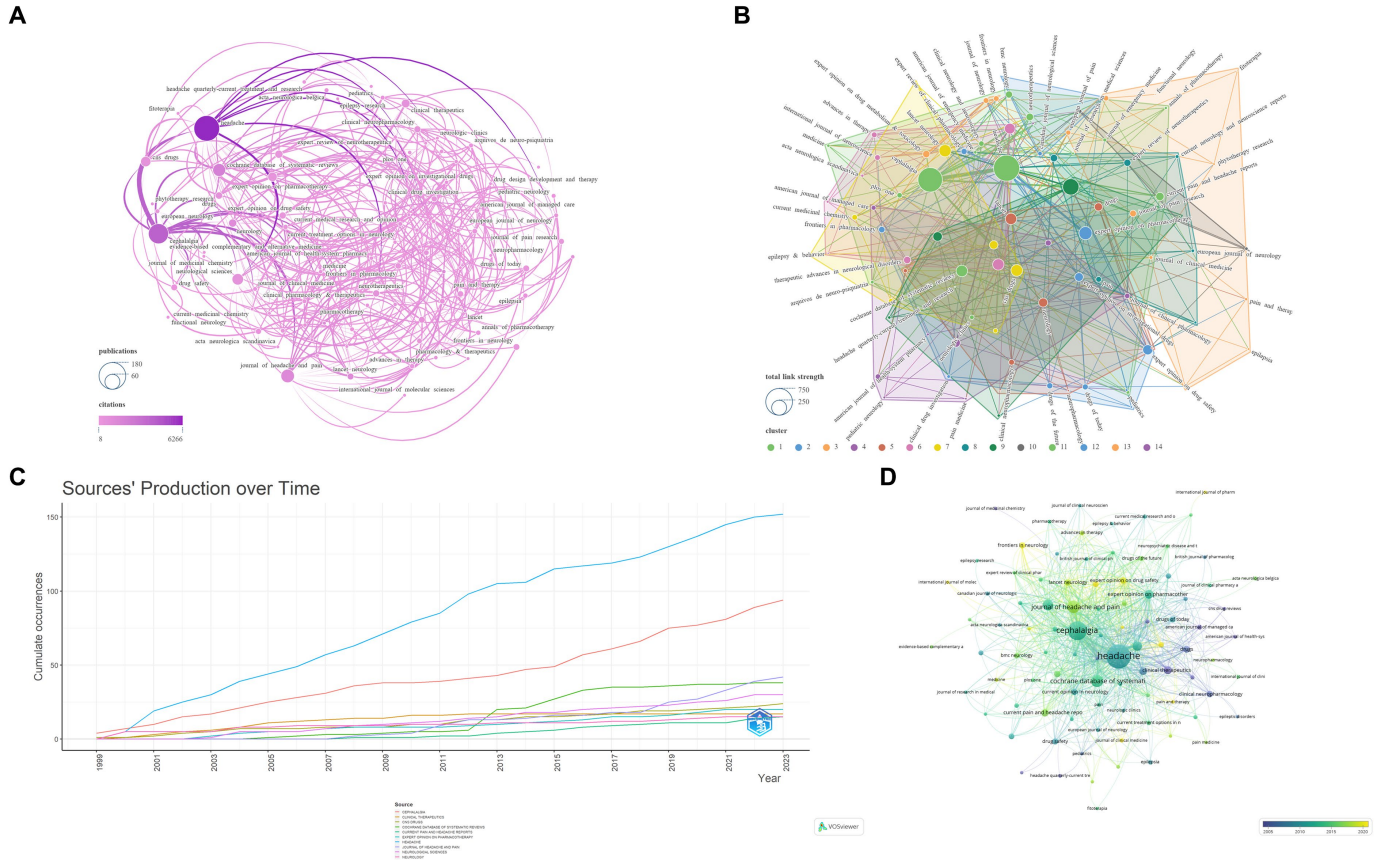
Network visualization of the Journal that contributed to the papers (**A**: Total number of publications and citations; **B**: Total link strength and cluster; **C**: Journals’ production over time; **D**: Publication changes year by year. The larger the graph area, the more the number of individual units, and the thicker the lines between the two units, the stronger the correlation).

Cluster analysis is a method for grouping highly similar targets (20). The h-index, a commonly used bibliometric indicator, is positively correlated with the quantity of publications and total citations (21). In comparison, the g-index offers a more precise evaluation of the contribution of highly cited items (22). Journal Impact Factor (JIF) and JIF Quartile data are obtained from the 2022 Journal Citation Reports.^3^ This study encompassed an analysis of 379 journals. By setting the ‘Minimum number of documents of a source’ to 3 in VOSviewer, we selected 93 qualifying journals. Subsequently, a visual analysis was conducted using VOSviewer and Scimago Graphica, as illustrated in Figure 3. The analyzed journals were primarily divided into 14 clusters. Table 1 presents the top 10 journals based on NP. Sixty percent of the top 10 journals are positioned in Quartile 1 of the Journal Citation Reports. The journal with the highest NP is HEADACHE (*N* = 152), followed by CEPHALALGIA (*N* = 94). Similarly, for the highest NC, HEADACHE (*N* = 6,266) and CEPHALALGIA (*N* = 4,035) lead the list. NEUROLOGY (*N* = 65.67) secures the highest position for Average Citations, followed by COCHRANE DATABASE OF SYSTEMATIC REVIEWS (*N* = 50.92), CEPHALALGIA (*N* = 42.93), and HEADACHE (41.22). The top three journals in terms of h-index and g-index are HEADACHE (*N* = 42, *N* = 71), CEPHALALGIA (*N* = 35, *N* = 61), and COCHRANE DATABASE OF SYSTEMATIC REVIEWS (*N* = 24, *N* = 38). Moreover, NEUROLOGY (*N* = 65.67) and COCHRANE DATABASE OF SYSTEMATIC REVIEWS (*N* = 50.92) are ranked first and second in terms of Average Citations. Similarly, NEUROLOGY (*N* = 9.9) and COCHRANE DATABASE OF SYSTEMATIC REVIEWS (*N* = 8.4) are the leading journals in JIF rankings. In terms of total link strength, HEADACHE (*N* = 659) ranks first, followed by CEPHALALGIA (*N* = 581) and JOURNAL OF HEADACHE AND PAIN (*N* = 258).

**Table 1:** The top 10 most productive journals.

Rank	Journals	h_index	g_index	NC	NP	Average citations	Total link strength	JIF	JIF quartile
1	Headache	42	71	6,266	152	41.22	659	5.0	Q1
2	Cephalalgia	35	61	4,035	94	42.93	581	4.9	Q1
3	Journal of headache and pain	21	34	1,206	42	28.71	258	7.4	Q1
4	Cochrane database of systematic reviews	24	38	1935	38	50.92	129	8.4	Q1
5	Neurological sciences	14	21	491	30	16.37	110	3.3	Q2
6	CNS drugs	13	21	482	24	20.08	132	6.0	Q1
7	Expert opinion on pharmacotherapy	11	17	301	20	15.05	166	3.2	Q3
8	Clinical therapeutics	11	17	481	17	28.29	75	3.2	Q3
9	Neurology	14	15	985	15	65.67	80	9.9	Q1
10	Current pain and headache reports	7	9	100	15	6.67	67	3.7	Q2

**Figure 4:**
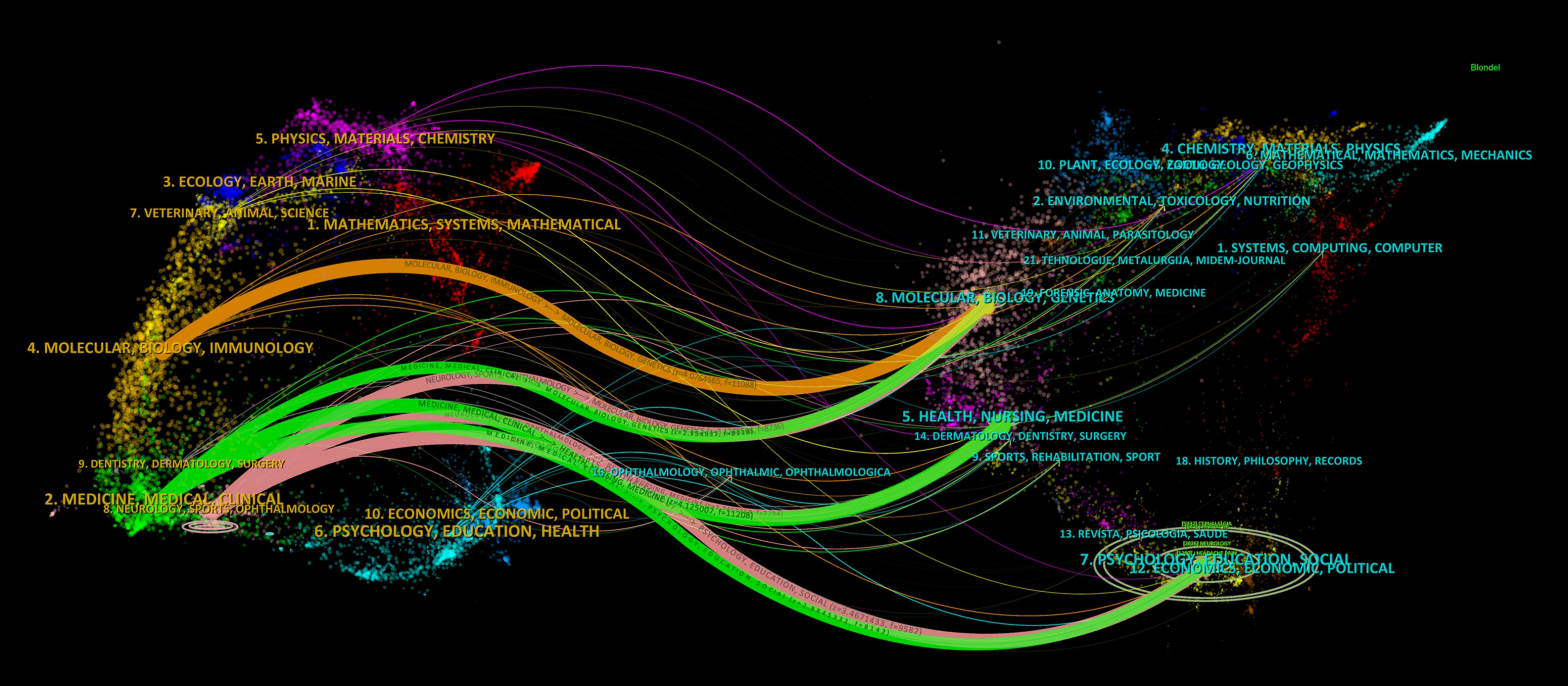
CiteSpace-based dual map overlay of journals.

As shown in Figure 4. The left part of the dual journal map represents the citing map, while the right part represents the cited map. The connecting lines between the two parts illustrate the citation relationships between journals and co-cited journals (23). The citing literature primarily originates from three fields, namely, MOLECULAR/BIOLOGY/IMMUNOLOGY, MEDICINE/MEDICAL/CLINICAL, and NEUROLOGY/SPORTS/OPHTHALMOLOGY, citing literature from seven paths in the fields of MOLECULAR/BIOLOGY/GENETICS, HEALTH/NURSING/MEDICINE, and PSYCHOLOGY/EDUCATION/SOCIAL.

### 3.4 Author analysis

**Figure 5:**
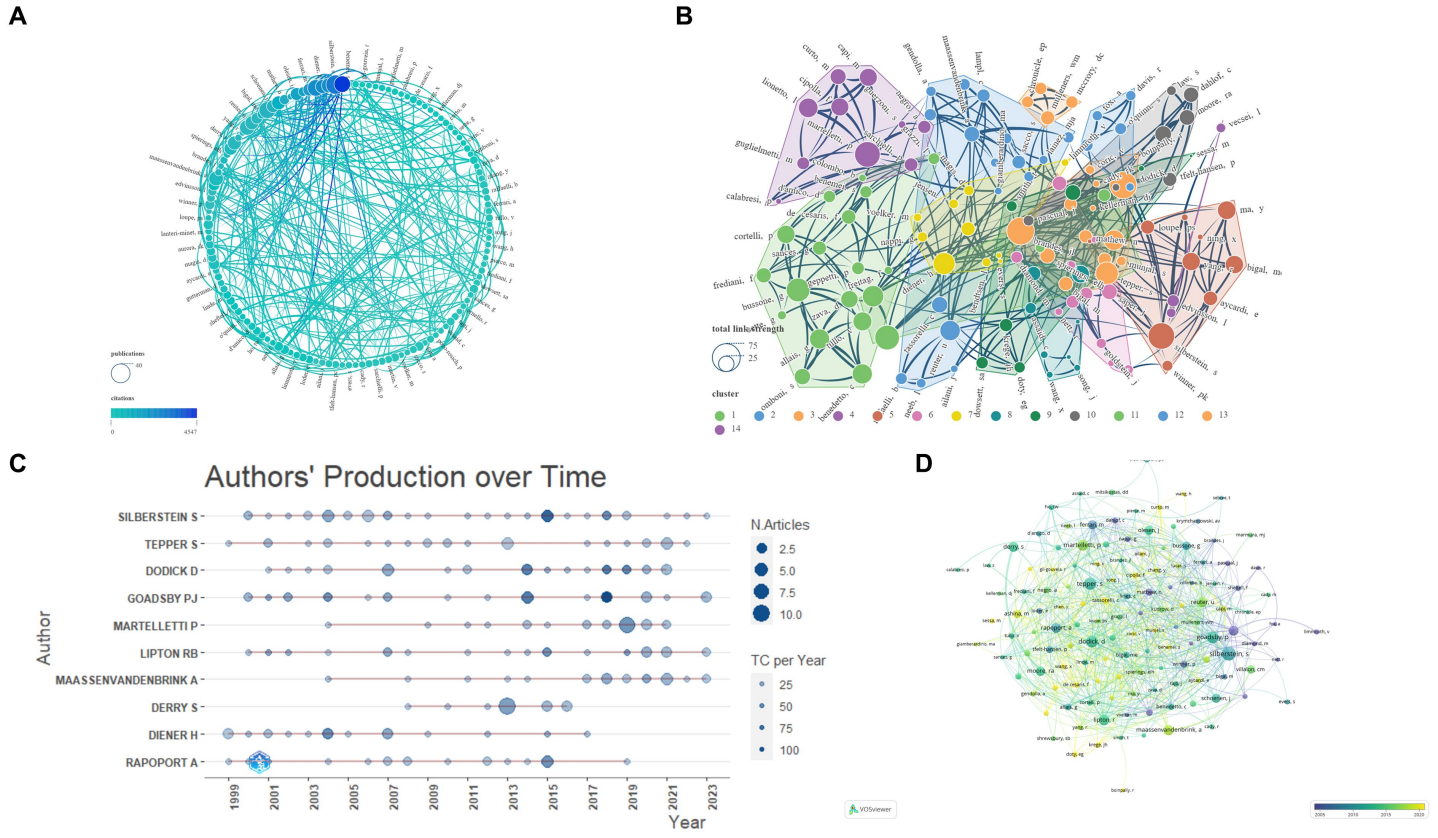
Network visualization of Authors that contributed to the papers (**A**: Total number of publications and citations; **B**: Total link strength and cluster; **C**: Authors’ production over time; **D**: Publication changes year by year. The larger the graph area, the more the number of individual units, and the thicker the lines between the two units, the stronger the correlation).

This study involves an extensive analysis of 4,235 authors. We consolidated similar author names, such as Silberstein, SD and Silberstein, S, and by setting the ‘Minimum number of documents of an article’ to 4 in VOSviewer, we ultimately identified 142 eligible authors. Subsequently, we conducted a visual analysis using VOSviewer and Scimago Graphica, as depicted in Figure 5. The analyzed journals were primarily categorized into 13 clusters. Table 2 presents the top 10 authors based on NP. The author with the highest NP is Silberstein, S (*N* = 37), followed by Tepper, S (*N* = 28), Dodick, D (*N* = 27), and Goadsby, PJ (*N* = 27). For the highest NC, Goadsby, PJ (*N* = 4,547) ranks first, followed by Silberstein, S (*N* = 3,224), and Lipton, RB (*N* = 2,635). Goadsby, PJ (*N* = 168.41) secures the top position for Average Citations, followed by Diener, H (*N* = 133.84) and Lipton, RB (*N* = 119.77). Silberstein, S (*N* = 23) leads in terms of the highest H-index, followed by Dodick, D (*N* = 21), Tepper, S (*N* = 19), and Goadsby, PJ (*N* = 19). Silberstein, S (*N* = 37) also holds the highest G-index, followed by Tepper, S (*N* = 28), Dodick, D (*N* = 27), and Goadsby, PJ (*N* = 27). In terms of total link strength, Lipton, RB (*N* = 66) ranks first, followed by Dodick, D (*N* = 64), and Silberstein, S (*N* = 61).

**Table 2:** The top 11 most productive journals.

Rank	Authors	h_index	g_index	NC	NP	Average citations	Total link strength
1	Silberstein S	23	37	3,224	37	87.14	61
2	Tepper S	19	28	1,002	28	35.79	45
3	Dodick D	21	27	2,530	27	93.7	64
4	Goadsby PJ	19	27	4,547	27	168.41	53
5	Martelletti P	15	24	797	24	33.21	57
6	Lipton RB	16	22	2,635	22	119.77	66
7	Maassenvandenbrink A	12	21	577	21	27.48	21
8	Derry S	15	19	707	19	37.21	24
9	Diener H	19	19	2,543	19	133.84	42
10	Rapoport A	15	19	1,135	19	59.74	36
11	Reuter U	13	19	756	19	39.79	36

### 3.5 Country/region and institution analysis

**Figure 6:**
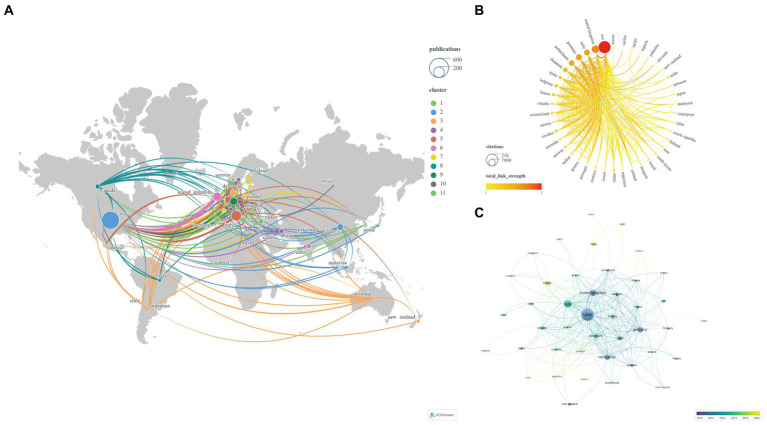
Networks showing the collaboration among Countries/regions in the papers (**A**: Total number of publications and clusters; **B**: Total link strength and citations; **C**: Publication changes year by year. The larger the graph area, the more the number of individual units, and the thicker the lines between the two units, the stronger the correlation).

This study encompasses 60 countries/regions. To ensure the reliability of our findings, we amalgamated certain country names, such as combining England, North Ireland, Scotland, and Wales into the United Kingdom. Subsequently, employing VOSviewer and Scimago Graphica, we visually analyzed 47 key countries, divided into 11 clusters, selected using VOSviewer with the setting ‘Minimum number of documents of a country = 2’ (Figure 6). The United States has established the most extensive national collaborative network, with a total link strength of 227, spanning 32 countries/regions. Table 3 displays the top 10 countries/regions based on NP ranking. The country with the highest NP is the United States (*N* = 481), followed by Italy (*N* = 163) and the United Kingdom (*N* = 117). The USA (*N* = 20,967) also leads in NC, followed by the United Kingdom (*N* = 8,353) and Germany (*N* = 5,266). The United Kingdom boasts the highest average citations (*N* = 71.39), followed by the Netherlands (*N* = 59.68) and Germany (*N* = 58.51).

**Table 3:** The top 10 most productive countries/regions and institutions.

Rank	1	2	3	4	5	6	7	8	9	10
Countries/Regions	USA	ITALY	UNITED KINGDOM	GERMANY	NETHERLANDS	DENMARK	CHINA	SPAIN	TURKEY	BELGIUM
NC	20,967	4,900	8,353	5,266	4,357	3,300	1,053	1,492	802	1,634
NP	481	163	117	90	73	65	64	50	43	37
Average citations	43.59	30.06	71.39	58.51	59.68	50.77	16.45	29.84	18.65	44.16
Total Link Strength	227	128	158	126	122	91	15	78	29	71
Links	32	27	26	22	25	22	10	22	14	20
										
Institutions	UNIV COPENHAGEN	ALBERT EINSTEIN COLL MED	MAYO CLIN	THOMAS JEFFERSON UNIV	ELI LILLY & CO	LEIDEN UNIV	UNIV CALIF LOS ANGELES	UNIV OXFORD	KINGS COLL LONDON	UNIV LIEGE
NC	2,586	2,858	1921	1824	1,016	2,167	802	707	1,443	1,098
NP	55	28	25	24	22	21	20	20	19	17
Average citations	47.02	102.07	76.84	76.00	46.18	103.19	40.10	35.35	75.95	64.59
Total Link Strength	46	57	51	26	27	27	25	2	37	17
Links	33	29	25	15	18	20	13	2	21	13

**Figure 7:**
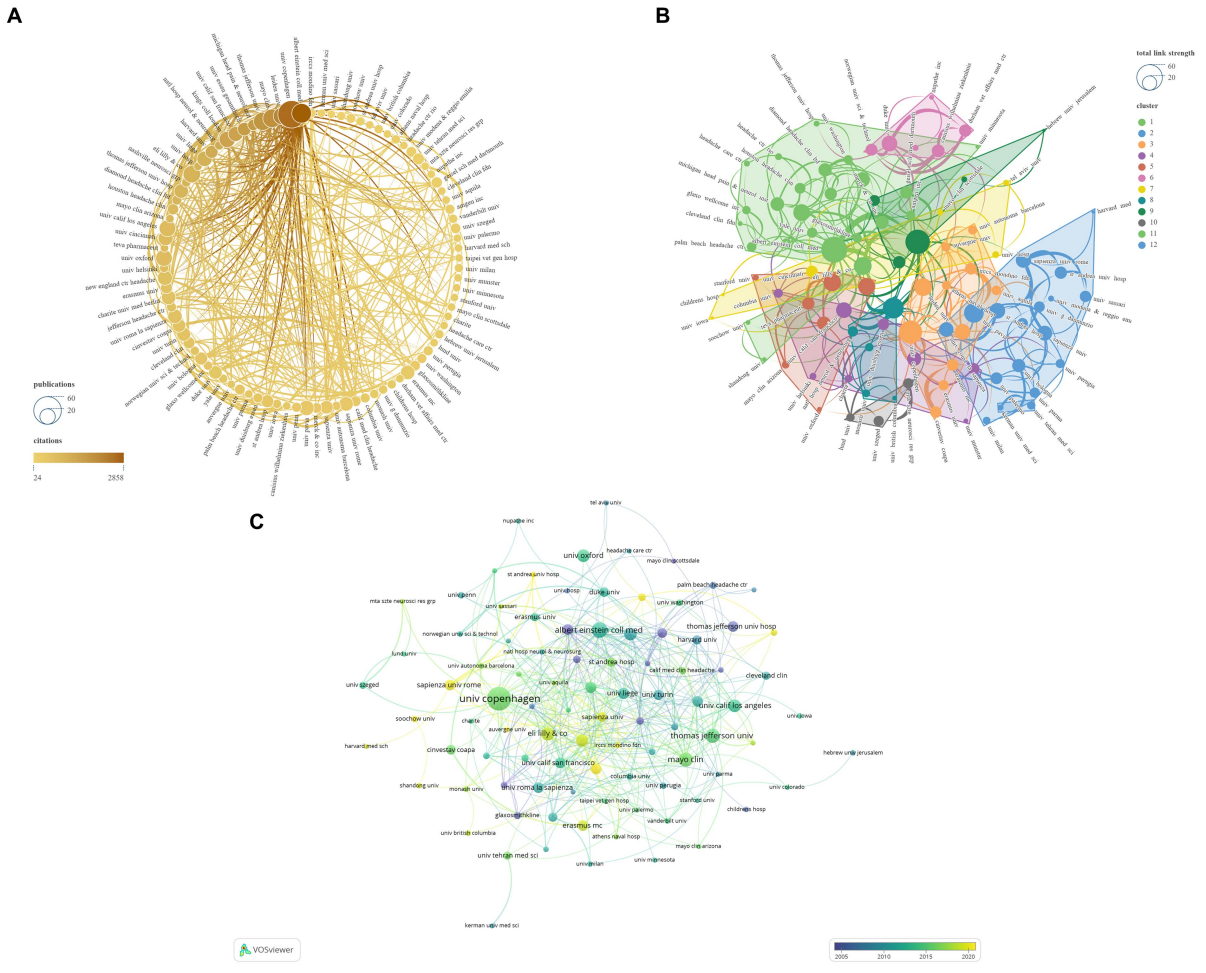
Network visualization of the Institutions that contributed to the papers (**A**: Total number of publications and citations; **B**: Total link strength and cluster; **C**: Publication changes year by year. The larger the graph area, the more the number of individual units, and the thicker the lines between the two units, the stronger the correlation).

Subsequently, we applied a ‘Minimum number of documents of an organization = 5’ setting to filter 103 institutions from a total of 1726, categorizing them into 12 clusters (Figure 7). Table 3 illustrates the top 10 institutions based on NP ranking. The institution with the highest NP is Univ Copenhagen (*N* = 55), followed by Albert Einstein Coll Med (*N* = 28) and Mayo Clinic (*N* = 25). The institution leading in NC is Albert Einstein Coll Med (*N* = 2,858), followed by Univ Copenhagen (*N* = 2,586) and Leiden Univ (*N* = 2,167). Leiden Univ boasts the highest average citations (*N* = 103.19), followed closely by Albert Einstein Coll Med (*N* = 102.07) and Mayo Clinic (*N* = 76.84). Albert Einstein Coll Med (*N* = 57) and Univ Copenhagen (*N* = 33) demonstrate the highest Total Link Strength and Links, respectively. Notably, Univ Oxford exhibits the lowest Total Link Strength (*N* = 2) and Links (*N* = 2) among the top 10 institutions.

### 3.6 Research direction

In the field, the literature can be categorized into various research directions based on Web of Science Categories. The most prominently observed categories are Clinical Neurology (*N* = 591), Pharmacology Pharmacy (*N* = 335), and Neurosciences (*N* = 306), garnering significant attention.

### 3.7 Keyword analysis

**Figure 8:**
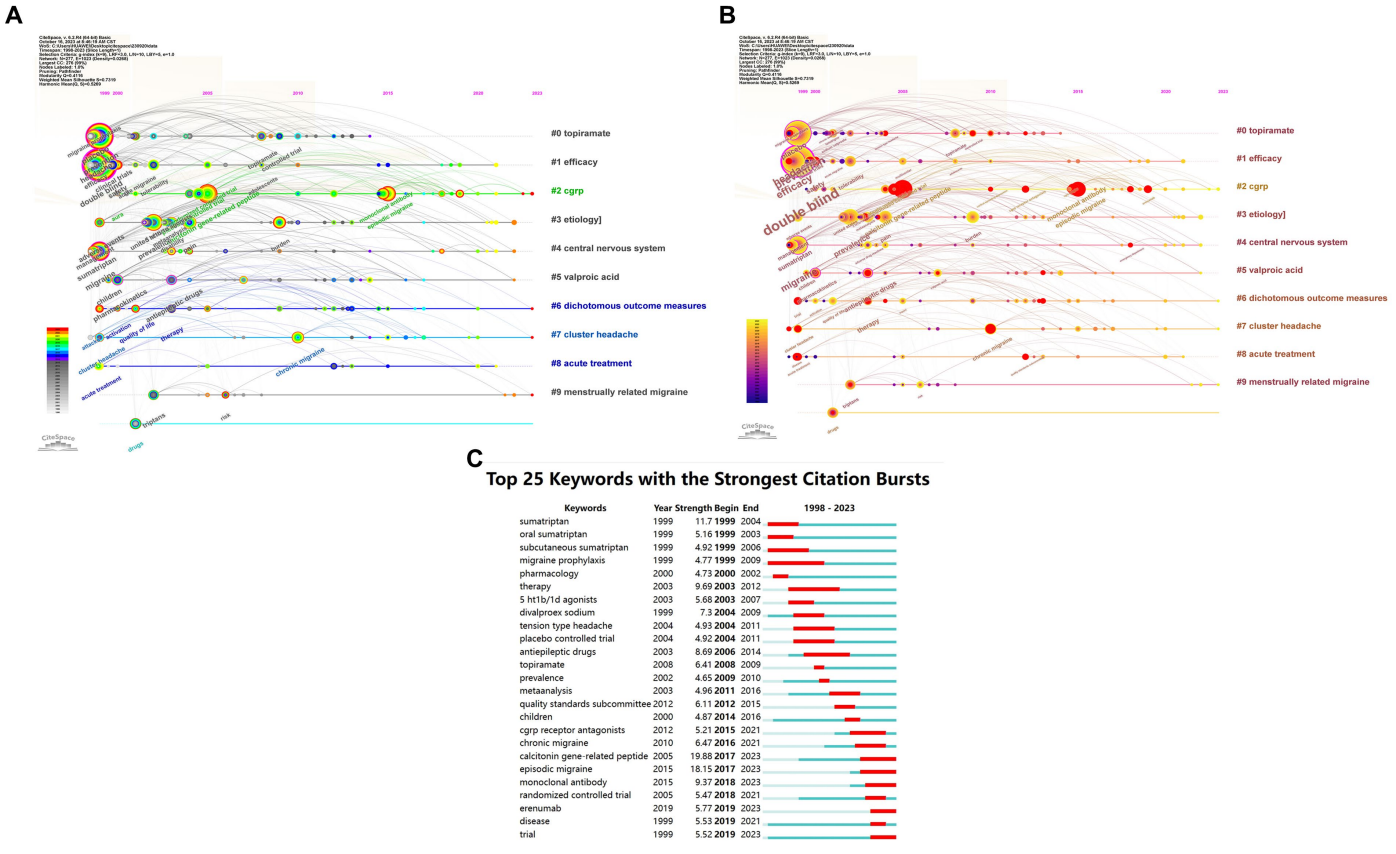
Network visualization of the Keyword clustering analysis of the papers changes by year and Keyword Burstiness (**A**: Keyword clustering analysis of the papers changes by year; **B**: Dynamically evolving high burstiness keywords change by year; **C**: Network visualization of the Keywords with the strongest citation bursts of the papers. The larger the graph area, the more the number of individual units, and the thicker the lines between the two units, the stronger the correlation).

Citespace has been utilized for keyword co-occurrence and clustering analysis. Co-occurrence analysis is a method that examines the frequency and patterns of the simultaneous occurrence of different research subjects (24). Burstiness describes a phenomenon where the frequency of appearance of a particular research subject significantly increases within a specific time frame (25). Among all 2,363 keywords, we merged those with similar meanings, such as ‘cgrp’ and ‘calcitonin gene-related peptide’, ‘prevention’ and ‘preventive treatment’, and others. Subsequently, using a scale factor k = 9 in Citespace, we finally filtered 277 keywords for analysis. The obtained data are evaluated as shown in Figure 8 and Table 4. Keywords representing research areas such as migraine are excluded from the scope of analysis. The most frequently occurring keywords are ‘double blind’ (*N* = 352), ‘prevention’ (*N* = 210), ‘efficacy’ (*N* = 190), ‘headache’ (*N* = 189), ‘calcitonin gene-related peptide’ (*N* = 128), ‘prevalence’ (*N* = 102), ‘placebo’ (*N* = 94), ‘safety’ (*N* = 84), ‘episodic migraine’ (*N* = 84), and ‘sumatriptan’ (*N* = 82). The most common types of migraine are ‘episodic migraine’ (*N* = 84), ‘chronic migraine’ (*N* = 50), and ‘cluster headache’ (*N* = 28). The most prevalent drug types include ‘triptans’ (*N* = 43), ‘antiepileptic drugs’ (*N* = 42), and ‘CGRP receptor antagonists’ (*N* = 21). The frequently encountered drugs are ‘sumatriptan’ (*N* = 82), ‘topiramate’ (*N* = 37), ‘sodium valproate’ (*N* = 19), ‘divalproex sodium’ (*N* = 17), ‘lamotrigine’ (*N* = 17), and ‘erenumab’ (*N* = 16). The targeted entities primarily consist of ‘calcitonin gene-related peptide’ (*N* = 128), ‘valproic acid’ (*N* = 17), and ‘CGRP receptor’ (*N* = 12). Subsequently, we conducted cluster analysis and visualization using the log-likelihood ratio algorithm. As the cluster map (Q = 0.4116, S = 0.7319) indicated Q > 0.3 and S > 0.5, the clustering quality was deemed satisfactory (12). These keywords were mainly divided into 10 clusters: Cluster #0 ‘topiramate’, Cluster #1 ‘efficacy’, Cluster #2 ‘CGRP’, Cluster #3 ‘etiology’, Cluster #4 ‘central nervous system’, Cluster #5 ‘valproic acid’, Cluster #6 ‘dichotomous outcome measures’, Cluster #7 ‘cluster headache’, Cluster #8 ‘acute treatment’, and Cluster #9 ‘menstrually related migraine’.

**Table 4:** The top 10 keywords and clusters of keywords cooccurring in the papers.

Rank	Keywords	Count	Centrality	Year	Cluster ID	Cluster name	Size	Mean (Year)	LLR
1	Double blind	352	0.24	1999	0	topiramate	39	2005	topiramate (23.42, 1.0E-4); valproate (17.49, 1.0E-4); migraine prophylaxis (16.2, 1.0E-4); propranolol (14, 0.001); divalproex sodium (12.41, 0.001)
2	Prevention	210	0.12	1999	1	efficacy	33	2004	efficacy (37.39, 1.0E-4); double blind (23.44, 1.0E-4); pharmacokinetic interaction (12.37, 0.001); sumatriptan nasal spray (12.37, 0.001); oral sumatriptan (10.54, 0.005)
3	Efficacy	190	0.06	1999	2	cgrp	33	2014	cgrp (42.97, 1.0E-4); monoclonal antibodies (31.99, 1.0E-4); gepants (26.08, 1.0E-4); cgrp receptor (26.08, 1.0E-4); monoclonal antibody (21.66, 1.0E-4)
4	Headache	189	0.1	1999	3	etiology	32	2008	etiology [(23.93, 1.0E-4); prevalence (17.8, 1.0E-4); united states (14.68, 0.001); migraine therapy (7.96, 0.005); adverse effects] (7.96, 0.005)
5	Migraine	158	0.19	1999	4	central nervous system	30	2007	central nervous system (12.8, 0.001); asthma (12.8, 0.001); pharmacovigilance (12.32, 0.001); adverse drug reaction (11.52, 0.001); prophylaxis (10.34, 0.005)
6	Calcitonin gene-related peptide	128	0.1	2005	5	valproic acid	29	2009	valproic acid (45.54, 1.0E-4); epilepsy (13.84, 0.001); antiepileptic drugs (11.91, 0.001); anticonvulsant (10.96, 0.001); epilepsy [drug therapy] (10.19, 0.005)
7	Prevalence	102	0.07	2002	6	dichotomous outcome measures	24	2009	dichotomous outcome measures (21.48, 1.0E-4); individual patient metaanalysis (16.52, 1.0E-4); intensity (16.52, 1.0E-4); migraine (14.43, 0.001); quality of life (14.17, 0.001)
8	Placebo	94	0.18	1999	7	cluster headache	21	2011	cluster headache (29.06, 1.0E-4); therapeutic use (15.89, 1.0E-4); neurostimulation (13.11, 0.001); transcranial magnetic stimulation (11.65, 0.001); transcranial direct current stimulation (11.65, 0.001)
9	Episodic migraine	84	0.02	2015	8	acute treatment	16	2006	acute treatment (31.25, 1.0E-4); low back pain (10.74, 0.005); ergotamine tartrate (7.94, 0.005); intravenous ketorolac (7.24, 0.01); pai (7.24, 0.01)
10	Safety	84	0.09	2000	9	menstrually related migraine	11	2010	menstrually related migraine (14.67, 0.001); bibn4096bs (10.9, 0.001); rat (7.32, 0.01); short-term prevention (7.32, 0.01); drug-genotype interactions (7.32, 0.01)

Based on the temporal dynamics of the identified keywords, it was observed that during the period from 1999 to approximately 2010, all 10 major clusters received significant attention. Notably, several prominent keywords during this time frame included “double blind,” “prevention,” “efficacy,” “headache,” “migraine,” “calcitonin gene-related peptide,” “prevalence,” “placebo,” “safety,” and “sumatriptan.” Subsequently, between 2010 and 2015, the attention toward Cluster #0 gradually diminished, accompanied by the emergence of keywords such as “episodic migraine,” “monoclonal antibody,” “CGRP receptor antagonists,” “quality standards subcommittee,” and “American Academy.” In the period spanning 2015 to 2020, significant attention was drawn toward keywords like “Erenumab,” “emergency department,” “AMG 334,” “ubrogepant,” and “blood–brain barrier.” As of 2023, researchers have directed their focus toward Cluster #2, Cluster #6, Cluster #7, and Cluster #9, with an emphasis on keywords such as “questionnaire,” “5-HT1F receptor agonist,” and “amylin.”

Subsequently, a burstiness analysis was conducted, where nodes marked with red circles indicated a higher level of burstiness. The size of the nodes was proportional to the magnitude of their burstiness. Notably, the burstiness analysis of the keywords revealed that “calcitonin gene-related peptide” (*N* = 19.88), “episodic migraine” (*N* = 18.15), and “sumatriptan” (*N* = 11.7) exhibited the highest levels of burstiness. Furthermore, the current persistent bursty keywords encompass “calcitonin gene-related peptide” (*N* = 19.88), “episodic migraine” (*N* = 18.15), “monoclonal antibody” (*N* = 9.37), “erenumab” (*N* = 5.77), and “trial” (*N* = 5.52).

### 3.8 Reference analysis

**Figure 9:**
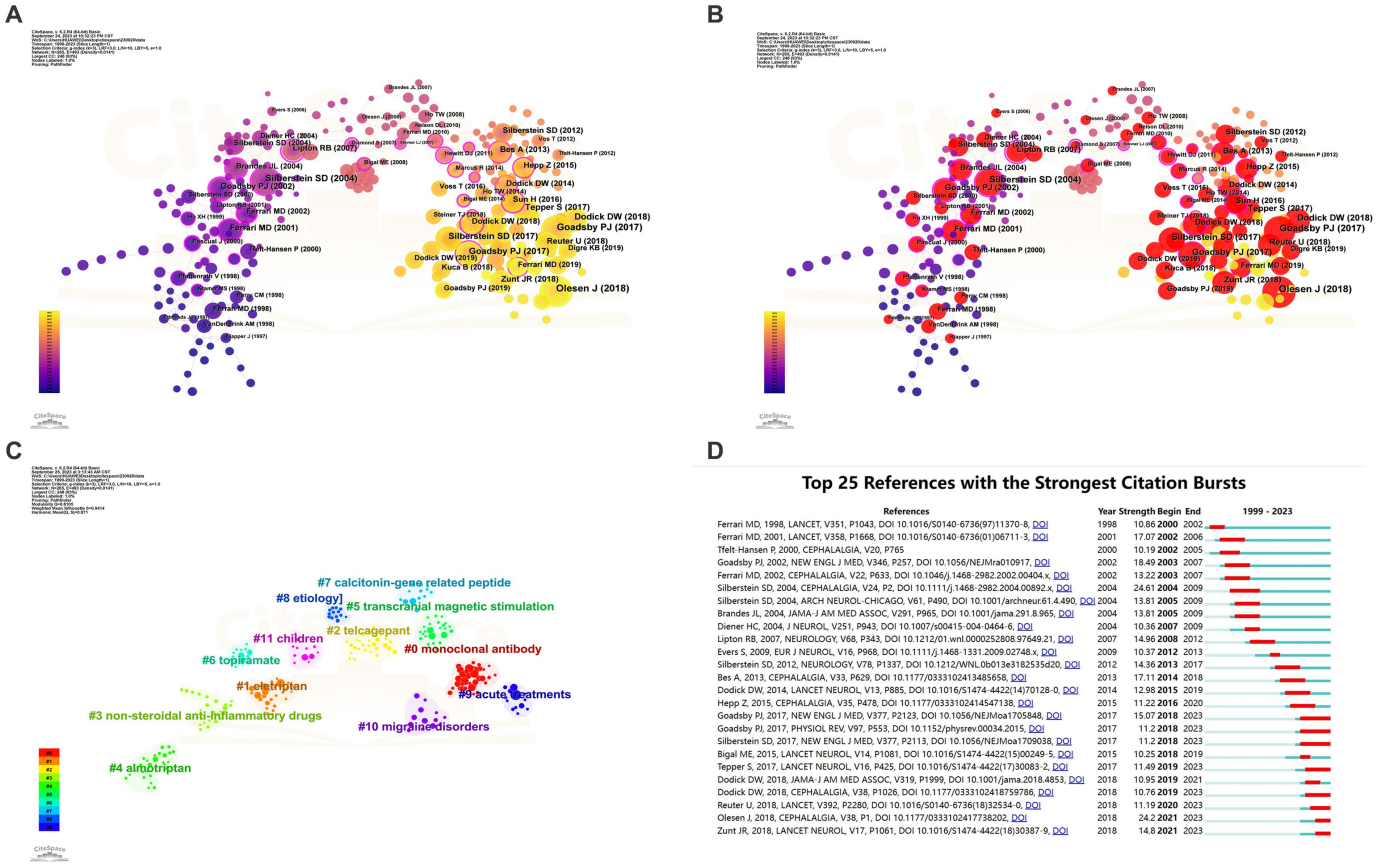
Network visualization of the Co-cited references clustering analysis of the papers changes by year and Co-cited references Burstiness (**A**: Network visualization of the co-occurring references; **B**: Network visualization of the high burstiness references; **C**: cluster analysis of references; **D**: Network visualization of the references with the strongest citation bursts of the papers. The larger the graph area, the more the number of individual units, and the thicker the lines between the two units, the stronger the correlation).

Citespace was employed for keyword co-occurrence and clustering analysis. Utilizing a scale factor k = 3 in Citespace, a meticulous screening process was conducted among 38,087 referenced articles, resulting in 265 articles that were included for analysis and subsequent visualization. The data obtained were evaluated as demonstrated in Figure 9 and Table 5. Notably, the most frequently cited references were the ‘Headache Classification Committee of the International Headache Society (IHS) The International Classification of Headache Disorders, 3rd edition’ (*N* = 67), followed by ‘A Controlled Trial of Erenumab for Episodic Migraine’ (*N* = 56), and ‘Migraine Pathophysiology and Its Clinical Implications’ (*N* = 45). The log-likelihood ratio algorithm was employed for the clustering analysis, revealing 12 principal clusters in the field, as indicated in the cluster map (Q = 0.8105, S = 0.9414): Cluster #0 Monoclonal Antibody, Cluster #1 Eletriptan, Cluster #2 Telcagepant, Cluster #3 Non-Steroidal Anti-Inflammatory Drugs, Cluster #4 Almotriptan, Cluster #5 Transcranial Magnetic Stimulation, Cluster #6 Topiramate, Cluster #7 Calcitonin-Gene Related Peptide, Cluster #8 Etiology, Cluster #9 Acute Treatments, Cluster #10 Migraine Disorders, and Cluster #11 Children.

**Table 5:** The top 12 references and clusters of cooccurring references in the papers.

Rank	1	2	3	4	5	6	7	8	9	10	11	12
Cited Reference	Headache Classification Committee of the International Headache Society (IHS) The International Classification of Headache Disorders, 3rd edition	A Controlled Trial of Erenumab for Episodic Migraine	Migraine pathophysiology and its clinical implications	Fremanezumab for the Preventive Treatment of Chronic Migraine	Pathophysiology of Migraine: A Disorder of Sensory Processing	ARISE: A Phase 3 randomized trial of erenumab for episodic migraine	Safety and efficacy of erenumab for preventive treatment of chronic migraine: a Randomized, double-blind, placebo-controlled phase 2 trial	Global, regional, and national burden of meningitis, 1990–2016: a systematic analysis for the Global Burden of Disease Study 2016	Migraine--current understanding and treatment	Efficacy and tolerability of erenumab in patients with episodic migraine in whom two-to-four previous preventive treatments were unsuccessful: a Randomized, double-blind, placebo-controlled, phase 3b study	Oral triptans (serotonin 5-HT(1B/1D) agonists) in acute migraine treatment: a meta-analysis of 53 trials	The International Classification of Headache Disorders, 3rd edition (beta version)
Authors	Olesen J	Goadsby PJ	Silberstein SD	Silberstein SD	Goadsby PJ	Dodick DW	Tepper S	Zunt JR	Goadsby PJ	Reuter U	Ferrari MD	Bes A
Journals	CEPHALALGIA	The New England journal of medicine	CEPHALALGIA	The New England journal of medicine	Physiological reviews	CEPHALALGIA	The Lancet. Neurology	The Lancet. Neurology	The New England journal of medicine	LANCET	LANCET	CEPHALALGIA
Count	67	56	45	42	42	41	37	33	32	32	31	30
brust	24.2	15.07	24.61	11.2	11.2	10.76	11.49	14.8	18.49	11.19	17.07	17.11
Centrality	0.06	0.01	0.43	0.06	0.1	0.08	0.06	0	0.4	0.08	0.3	0.17
Year	2018	2017	2004	2017	2017	2018	2017	2018	2002	2018	2001	2013
												
Cluster ID	0	1	2	3	4	5	6	7	8	9	10	11
Cluster Name	monoclonal antibody	eletriptan	telcagepant	non-steroidal anti-inflammatory drugs	almotriptan	transcranial magnetic stimulation	topiramate	calcitonin-gene related peptide	etiology	acute teatments	migraine disorders	children
Size	35	31	28	26	25	24	14	14	13	13	13	12
Mean (Year)	2018	2002	2008	1999	1997	2013	2003	2014	2010	2019	2018	2006
Label (LLR)	monoclonal antibody (22.92, 1.0E-4); cgrp (14.5, 0.001); erenumab (10.61, 0.005); drug safety (6.39, 0.05); eptinezumab (6.39, 0.05)	eletriptan (17.22, 1.0E-4); menstrually related migraine (8.58, 0.005); bibn4096bs (8.58, 0.005); topiramate (7.89, 0.005); side effects (5.01, 0.05)	telcagepant (21.26, 1.0E-4); cgrp receptor antagonists (15.9, 1.0E-4); pediatric migraine (6.91, 0.01); adolescents (6.91, 0.01); divalproex sodium (5.33, 0.05)	non-steroidal anti-inflammatory drugs (8.29, 0.005); triptans with enhanced lipophilicity (tels) (8.29, 0.005); migraine management (8.29, 0.005); almotriptan malate (5.55, 0.05); drugs - availability (5.55, 0.05)	almotriptan (14.8, 0.001); ergotamine (11.03, 0.001); triptans (9.3, 0.005); serotonin agonists (7.35, 0.01); pharmacokinetics (5.75, 0.05)	transcranial magnetic stimulation (9.25, 0.005); cluster headache (7.59, 0.01); cgrp (6.16, 0.05); cefaly (4.62, 0.05); transcutaneous supraorbital neurostimulation (4.62, 0.05)	topiramate (10.46, 0.005); antiepileptic (6.7, 0.01); responder rate (6.7, 0.01); Randomized trial (6.7, 0.01); chronic migraine treatment (6.7, 0.01)	calcitonin-gene related peptide (10.37, 0.005); drug development (10.37, 0.005); bbb (5.17, 0.05); neurovascular disorder (5.17, 0.05); cell surface g protein receptors (5.17, 0.05)	etiology] (36.67, 1.0E-4); therapeutic use] (30.46, 1.0E-4); adult (18.16, 1.0E-4); humans (18.16, 1.0E-4); migraine (12.24, 0.001)	acute treatments (4.5, 0.05); monoclonal (4.5, 0.05); onabotulinum toxin a (4.5, 0.05); rct (4.5, 0.05); pharmaceutical preparations (4.5, 0.05)	migraine disorders (9.56, 0.005); acute treatment (7.54, 0.01); rimegepant (5.61, 0.05); cgrp (4.94, 0.05); abortive treatment (4.6, 0.05)	children (7.65, 0.01); neuromodulators (5.66, 0.05); music therapy (5.66, 0.05); neck (5.66, 0.05); norepinephrine reuptake inhibitor (5.66, 0.05)

Subsequently, we conducted an analysis of burstiness, revealing that the most explosively impactful literature included “Migraine Pathophysiology and Its Clinical Implications” (*N* = 24.61), followed by “Headache Classification Committee of the International Headache Society (IHS): The International Classification of Headache Disorders, 3rd Edition” (*N* = 24.2) and “Migraine - Current Understanding and Treatment” (*N* = 18.49). Eight publications have demonstrated sustained high burstiness to date, namely “Headache Classification Committee of the International Headache Society (IHS): The International Classification of Headache Disorders, 3rd Edition” (*N* = 24.2), “A Controlled Trial of Erenumab for Episodic Migraine” (*N* = 15.07), “Global, Regional, and National Burden of Meningitis, 1990–2016: A Systematic Analysis for the Global Burden of Disease Study 2016” (*N* = 14.8), “Safety and Efficacy of Erenumab for Preventive Treatment of Chronic Migraine: A Randomised, Double-blind, Placebo-controlled Phase 2 Trial” (*N* = 11.49), “Fremanezumab for the Preventive Treatment of Chronic Migraine” (*N* = 11.2), “Pathophysiology of Migraine: A Disorder of Sensory Processing” (*N* = 11.2), “Efficacy and Tolerability of Erenumab in Patients with Episodic Migraine in Whom Two-to-Four Previous Preventive Treatments Were Unsuccessful: A Randomised, Double-blind, Placebo-controlled, Phase 3b Study” (*N* = 11.19), and “ARISE: A Phase 3 Randomized Trial of Erenumab for Episodic Migraine” (*N* = 10.76).

## 4 Discussion

### 4.1 General information

This study represents the inaugural application of bibliometric analysis to investigate the developmental status and trends within the fields of Migraine and Drug-Related Side Effects and Adverse Reactions in the Web of Science Core Collection (WOSCC) from January 1, 1998, to August 31, 2023. Encompassing a corpus of 1,201 publications, the analysis spans 60 countries/regions, 379 journals, 4,235 authors, 1726 institutions, 2,363 keywords, and 38,142 references.

Based on the annual trends observed in the published literature and citations, a significant upsurge in citations is apparent, indicating a notable surge in the field’s attention since 1999. For the majority of the period between 1999 and 2018, the quantity of publications ranged between 30 and 40 articles, with notable peaks in 2012, 2013, and 2015, surpassing 50 articles. Notably, the years 2019 to 2021 exhibited a remarkable growth trajectory, sustaining a high level thereafter. As of our latest literature review, up until August 31, 2023, the field’s prominence continues to remain elevated, as indicated by the combined tally of 2,479 citations and 45 publications in the initial 8 months of the year (Figure 2).

In the realm of journal analysis, the prominent positions held by HEADACHE and CEPHALALGIA, ranking first and second in terms of NP, NC, total link strength, h-index, and g-index, alongside their commendable performance in other pertinent indicators, solidify their standing as the most influential journals in the field. Notably, NEUROLOGY and COCHRANE DATABASE OF SYSTEMATIC REVIEWS boast the highest average citations and JIF, underscoring the high caliber and widespread recognition of their publications. The annual publication trends of these journals underscore the recent dominance of HEADACHE and CEPHALALGIA in the field, while also highlighting the positive trajectory of the JOURNAL OF HEADACHE AND PAIN in recent years. Furthermore, the evolutionary relationships in journal publication over time suggest that journals like Frontiers in Neurology and Expert Opinion on Drug Safety might be emerging as novel forces in this domain (Figure 3; Table 1). The dual journal map delineates the intricate interrelations between citing and cited literature, indicating the multidisciplinary, multi-themed, and cross-disciplinary nature of the related research in this domain, necessitating researchers to possess a broad perspective and diverse knowledge base (Figure 4).

Concerning author analysis, taking all included metrics into account, it is apparent that SILBERSTEIN S and GOADSBY PJ wield significant influence in the field. Despite a lower NP count (*N* = 19), DIENER H secures the second-highest average citations (133.84), indicating widespread acclaim for the quality of his publications. LIPTON RB’s highest total link strength signifies a closely collaborative relationship with other researchers within the field. The annual publication trends of authors reveal the sustained impact exerted by SILBERSTEIN S, GOADSBY PJ, LIPTON RB, and MAASSENVANDENBRINK A. Similarly, the evolutionary relationships in journal publication over time suggest that individuals such as TASSORELLI C and WANG X might be promising authors worthy of attention within the field (Figure 5; Table 2).

Analyses of the countries/regions reveal that the major impact-driving forces in this field are primarily concentrated in North America and Europe, where intricate and extensive networks of collaboration have been established. The collaborative network established by the United States encompasses the highest number of countries/regions, thus yielding the highest NP, NC, Total Link Strength, and Links, making it the most influential country in this field. The United Kingdom boasts the highest Average Citations and also holds high rankings in other relevant indicators, signifying widespread recognition of its high-quality publications and substantial influence in the field. Evolutionary trends over time in the publication relationships of countries/regions illustrate recent robust activity in the field by China, India, Argentina, among others (Figure 6; Table 3). UNIV Copenhagen, Albert Einstein College of Medicine, and Leiden University hold the top positions in NP, NC, and Average Citations rankings, respectively, placing them unequivocally as the most influential institutions in the field. Both Albert Einstein College of Medicine and UNIV Copenhagen have actively contributed to establishing broad and intricate networks of collaboration. Notably, Univ Oxford appears to require strengthened collaborations with other institutions. The evolving institutional publication relationships over time highlight Eli Lilly & Co, Sapienza University, and Erasmus MC as noteworthy institutions at the forefront of the field (Figure 7; Table 3).

### 4.2 Knowledge base and future perspectives

The most prevalent keywords underscore the meticulous demands of clinical research methods in this field, emphasizing the epidemiology and burden of migraine, with a focus on drug therapies and their characteristics. The assessment encompasses treatment efficacy, safety, and tolerability, while maintaining a keen interest in pain management and mechanistic research. The clustering of keywords highlights diverse research directions, including etiology, mechanisms, crucial targets, multiple drug approaches, efficacy evaluations, and various types of migraine. The keyword clustering underscores distinct research domains such as etiology, the pivotal role of calcitonin-gene related peptide as a key factor in this condition, and several drugs, including topiramate and valproic acid. Researchers have concentrated their attention on the central nervous system’s organizational structure, emphasizing the application of dichotomous outcome measures to enhance the reliability of efficacy assessments. Moreover, acute treatment and different types of migraines, such as cluster headaches and menstrually related migraines, have garnered significant attention within this field. Around 1999–2010, researchers primarily focused on the prevention, treatment efficacy, and safety of migraine, emphasizing the crucial role of the double-blind placebo in experimental design. Meanwhile, there was a gradual increase in the application of sumatriptan for migraine management, with growing attention on calcitonin gene-related peptide as a significant factor in migraine. Around 2010–2015, researchers gradually amplified their focus on episodic migraine, exhibiting strong interest in CGRP receptors, CGRP receptor antagonists, and monoclonal antibodies. The American Academy and Quality Standards Subcommittee emerged as the most influential academic entities in this field. Around 2015–2020, there was a gradual increase in reports related to three drugs targeting the CGRP system, namely erenumab, amg 334, and ubrogepant. The emergency department became a new focal point, and the critical role of the blood–brain barrier received further affirmation. As of 2023, there has been a significant emphasis on the role of questionnaires in evaluations. New research focuses on 5-ht1f receptor agonists, apoptosis, amylin, and antidepressants, among others. Burstiness analysis of the keywords demonstrates that researchers are particularly focused on trial methods, experimental design, efficacy assessment, and various types of drugs. Based on the current persistently impactful keywords, we predict that future research in this field may concentrate on episodic migraine, calcitonin gene-related peptide, and related drugs, aiming to find robust evidence through clinical trials (Figure 8; Table 4).

The most cited literature highlights researchers’ emphasis on the precise diagnosis of migraine types (3, 26), exploring the pathophysiological mechanisms of this condition, and the consequent development of various pharmacological treatments (27–29). Meningitis has garnered attention from researchers in the field (30). A meta-analysis involving 24,089 patients and 53 clinical trials demonstrated the effectiveness and good tolerability of all oral triptan class drugs (18). Five studies investigated the CGRP system-related drugs Erenumab and Fremanezumab. A 12-week study involving 1,130 patients revealed that compared to a placebo, Fremanezumab significantly reduced the frequency of headaches, with main adverse reactions being injection-site reactions primarily characterized by pain (31). Four studies confirmed the preventive and therapeutic effects of Erenumab on episodic and chronic migraines, as well as its potential as an ideal option for patients with difficult-to-treat migraines. The safety profile of Erenumab was found to be similar to the placebo across the four studies, with the most common adverse events being injection site pain, upper respiratory tract infections, nausea, and nasopharyngitis (32–35). Cluster analysis encompassed various aspects of the research, including etiology, the calcitonin-gene-related peptide as a key factor in migraines, the widely discussed monoclonal antibodies, and multiple drugs such as eletriptan, telcagepant, non-steroidal anti-inflammatory drugs, and topiramate. Transcranial magnetic stimulation, as a non-invasive and relatively safe therapy, has gained widespread application. Additionally, acute treatments and the special group of children have received special attention from researchers in the field. Burstiness analysis of references indicates that the precise diagnosis and pathophysiology of migraines, the role of meningitis, and the future potential of the drugs Erenumab and Fremanezumab are likely to continue to be of enduring interest among researchers in the field (Figure 9; Table 5).

**Figure 10:**
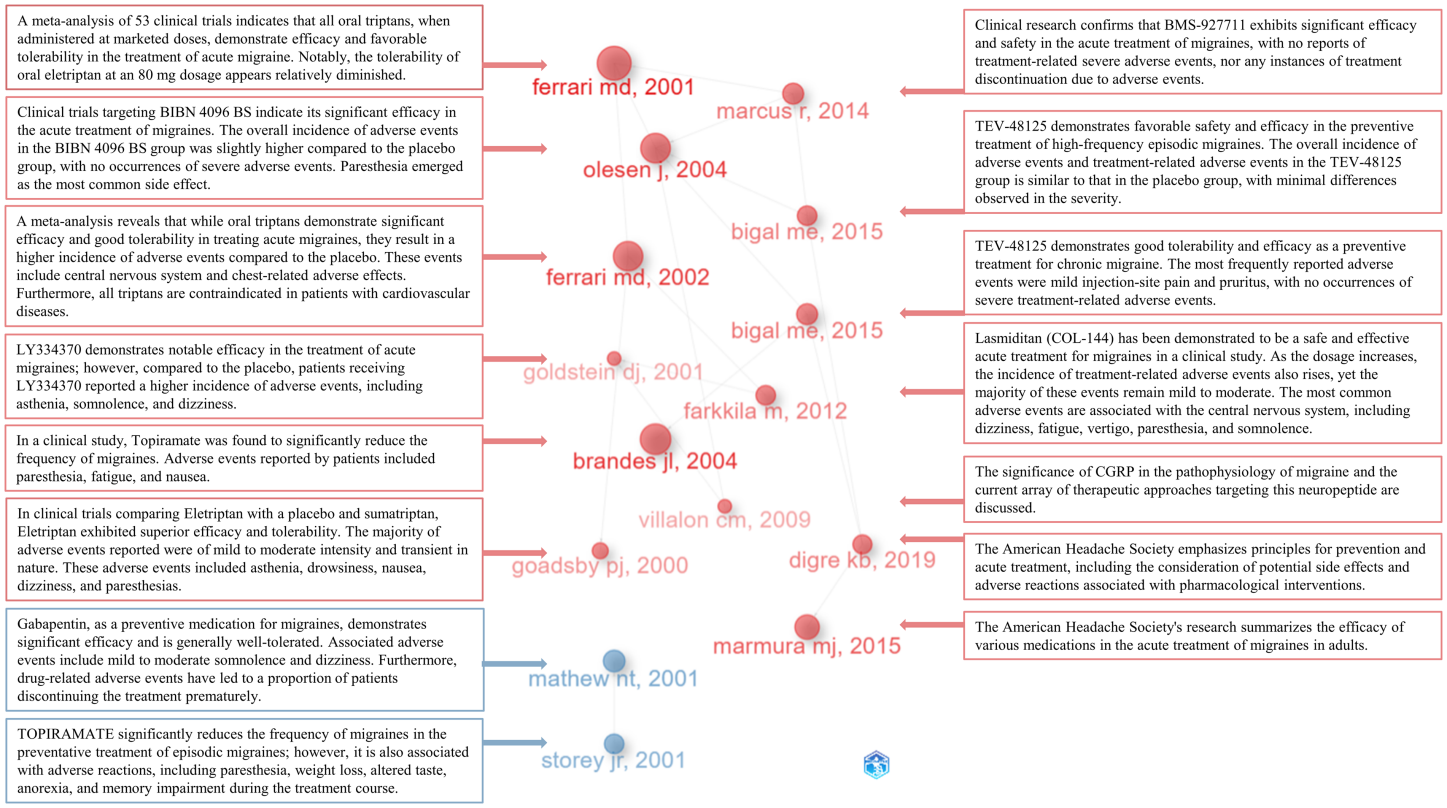
15 seminal publications and their principal contents.

In-depth understanding of the developmental trajectory in this field can be attained through a comprehensive analysis and interpretation of seminal literature, key terms, and results from reference bibliographies. Leveraging the historiographic analysis feature within the R package ‘bibliometrix,’ we identified a set of 15 seminal publications within this domain. Subsequently, we generated visualizations, annotating key insights extracted from these publications (Figure 10). By amalgamating the primary findings from these 15 publications, it becomes evident that the majority of research endeavors have been devoted to conducting high-quality clinical trials investigating the efficacy and safety of various pharmaceutical agents. These therapeutic agents encompass a diverse range, each exploiting distinct mechanisms of action. Notably, these include a multitude of triptan-class drugs such as Eletriptan and Sumatriptan, acting as SEROTONIN 5-HT1B/1D AGONISTS (36). Additionally, there are SELECTIVE SEROTONIN 1F (5-HT1F) RECEPTOR AGONIST compounds like LY334370 (37) and lasmiditan (38). Further inclusions consist of CALCITONIN GENE-RELATED PEPTIDE RECEPTOR ANTAGONISTS, exemplified by BIBN 4096 BS (17) and BMS-927711 (39). The repertoire also encompasses monoclonal anti-CGRP antibodies, notably TEV-48125 (40, 41), and antiepileptic drugs like Topiramate (19, 42) and gabapentin (43). Within the scope of these clinical investigations, therapeutic agents targeting acute migraine treatment are represented by Eletriptan, LY334370, BIBN 4096 BS, BMS-927711, and lasmiditan (17, 36–39). Furthermore, preventive measures for migraine comprise TOPIRAMATE and TEV-48125 (19, 40–42). Significantly, these pharmaceutical interventions have demonstrated remarkable efficacy and safety profiles, with the majority of adverse events being of mild to moderate severity. Notably, a comprehensive meta-analysis has corroborated the effectiveness and safety of the triptan-class medications available on the market (18, 44). The pivotal role of the CGRP system in migraine pathophysiology, as well as the heightened attention toward multiple CGRP RECEPTOR ANTAGONISTS, has been well-documented (45). Furthermore, authoritative reports from THE AMERICAN HEADACHE SOCIETY have underscored safety concerns in acute migraine therapy (7, 46).

In addition to seminal literature within the field, researchers have shown a keen interest in various types of pharmaceuticals, including the monoclonal anti-CGRP antibody [LY2951742 (47), Fremanezumab (31), Erenumab (32), Galcanezumab (48), ALD403 (49)], CGRP receptor antagonist [Ubrogepant (50)], NMDA receptor antagonist [Memantine (51)], antidepressant [Amitriptyline (52)], antiepileptic drugs [Valproate (53), Zonisamide (54)], another antidepressant [Venlafaxine (55)], nonsteroidal anti-inflammatory drug [diclofenac-potassium (56)], corticosteroid [Dexamethasone (57)], calcium channel blocker [Flunarizine (58)], and classic Chinese herbal remedies (59). Adverse reactions associated with these medications are generally mild to moderate, primarily including asthenia, drowsiness, nausea, dizziness, paresthesias, fatigue, vertigo, weight loss, altered taste, and anorexia. Moreover, researchers in clinical trials have focused on severe adverse drug reactions (60, 61), drug discontinuation events (62), the safety of combination therapies (63), the safety of medication for patients with underlying conditions (64), the safety of medication during specific physiological periods (65), migraine types associated with specific symptoms (66), various methods of administration (67–69), and the reporting of rare drug-related cases (70).

According to our analysis, drugs targeting the calcitonin gene-related peptide (CGRP) system undeniably represent a focal point in current migraine therapeutics. Two categories of drugs address the CGRP system: monoclonal anti-CGRP antibodies, including Erenumab, Fremanezumab, Galcanezumab, and Eptinezumab; and CGRP receptor antagonists, including Ubrogepant, Rimegepant, Atogepant, and Zavegepant. These drugs have demonstrated commendable clinical efficacy, and their safety profiles have been endorsed, with predominantly mild to moderate side effects. However, some studies have reported data inconsistent with clinical trials. For example, Erenumab may have a higher likelihood of causing constipation in patients and a higher discontinuation rate (71). Erenumab treatment appears to be significantly associated with hypertension (72). Furthermore, case reports have described drug-related myocardial infarction (73) and ocular myasthenia gravis-like symptoms (74). Galcanezumab, on the other hand, is considered to potentially have higher efficacy and similar tolerability (75). Additionally, various CGRP-targeting drugs are thought to increase the risk of alopecia (76). As these drugs have been approved for marketing only in recent years [especially Zavegepant, which was approved this year (77)], there is currently a lack of comprehensive reporting on drug-related side effects. The long-term effectiveness and safety of these drugs require further investigation. In the future, more detailed and reliable evidence for drug assessment can be facilitated through the implementation of long-term, cross-regional, multicenter, large-scale randomized controlled trials and real-world studies.

## 5 Limitations

Several limitations are present in this study. Firstly, only data from the WOS-SCIE database were included, ensuring the quality of evidence but possibly overlooking some relevant studies. To minimize subjective inclusion differences, we refrained from conducting further manual screening of the included literature, enhancing the objectivity of the analysis but potentially compromising the precision of the study. Non-English publications were excluded, potentially underestimating the impact of non-English scholarly contributions. Additionally, due to data constraints, publications beyond September 2023 were not incorporated.

## 6 Conclusion

In summary, our study synthesizes the knowledge base in the field of migraine and Drug-Related Side Effects and Adverse Reactions, while also forecasting the developmental trends in this domain. We conducted visual analyses of pivotal journals, authors, countries/regions, institutions, keywords, and references contributing to this field. Our findings suggest that researchers are likely to sustain their focus on the CGRP system and conduct research on 5-ht1f receptor agonists, apoptosis, amylin, and antidepressants, alongside the development of novel drugs based on monoclonal antibody technology. Continuous attention to the efficacy and safety of various prominent drugs, such as renumab and Fremanezumab, is crucial, given the wealth of new evidence continually provided by large-scale randomized controlled clinical trials. Further, it is imperative to conduct larger-scale evaluations of acute and preventive treatments for different populations and migraine types. Exploration into the precise diagnosis of migraine and its pathophysiological mechanisms, with a focus on central nervous system and meningeal tissue structures, is also recommended. To ensure high-quality evidence, we propose enhancing the application of dichotomous outcome measures and questionnaires in the assessment of clinical trial results.

## 7 Recommendations for healthcare professionals

1. Conduct large-scale, multi-center, double-blind, placebo-controlled, and randomized clinical trials targeting diverse regions, ethnicities, ages, and genders.
2. Assess the safety of medications during specific physiological phases such as the perimenstrual period, pregnancy, and menopause.
3. Intensify monitoring when medications are co-administered with commonly prescribed clinical drugs.
4. Strengthen observation and evaluation for patients with underlying medical conditions.
5. Enhance mechanistic research on adverse drug reactions.
6. Emphasize post-treatment follow-up and refine long-term drug assessments.
7. Investigate interactions between CGRP and other pivotal substances in migraine pathophysiology.

## Data availability statement

The raw data supporting the conclusions of this article will be made available by the authors, without undue reservation.

## Author contributions

SW: Conceptualization, Investigation, Software, Visualization, Writing – original draft. HL: Conceptualization, Investigation, Software, Visualization, Writing – original draft. DY: Investigation, Writing – review & editing. LZ: Investigation, Writing – review & editing. XL: Software, Visualization, Writing – review & editing. YN: Software, Visualization, Writing – review & editing. YT: Writing – original draft. XW: Writing – original draft. JH: Funding acquisition, Writing – review & editing.

## Footnotes

^1^ https://www.webofscience.com/wos/woscc/basic-search

^2^ https://www.bibliometrix.org

^3^ https://jcr.clarivate.com/jcr/browse-journals
